# Elimination of Flammable Gas Effects in Cerium Oxide Semiconductor-Type Resistive Oxygen Sensors for Monitoring Low Oxygen Concentrations

**DOI:** 10.3390/s150409427

**Published:** 2015-04-21

**Authors:** Toshio Itoh, Noriya Izu, Takafumi Akamatsu, Woosuck Shin, Yusuke Miki, Yasuo Hirose

**Affiliations:** 1 National Institute of Advanced Industrial Science and Technology (AIST), Shimo-Shidami, Moriyama-ku, Nagoya 463-8560, Japan; E-Mails: n-izu@aist.go.jp (N.I.); t-akamatsu@aist.go.jp (T.A.); w.shin@aist.go.jp (W.S.); 2 Taiyo Nippon Sanso Corporation, 10 Okubo, Tsukuba, Ibaraki 300-2611, Japan; E-Mails: Yusuke.Miki@tn-sanso.co.jp (Y.M.); Yasuo.Hirose@tn-sanso.co.jp (Y.H.)

**Keywords:** resistive oxygen sensor, cerium oxide, zirconium-doped cerium oxide, layered thick film, catalytic layer, platinum

## Abstract

We have investigated the catalytic layer in zirconium-doped cerium oxide, Ce_0.9_Zr_0.1_O_2_ (CeZr10) resistive oxygen sensors for reducing the effects of flammable gases, namely hydrogen and carbon monoxide. When the concentration of flammable gases is comparable to that of oxygen, the resistance of CeZr10 is affected by the presence of these gases. We have developed layered thick films, which consist of an oxygen sensor layer (CeZr10), an insulation layer (Al_2_O_3_), and a catalytic layer consisting of CeZr10 with 3 wt% added platinum, which was prepared *via* the screen printing method. The Pt-CeZr10 catalytic layer was found to prevent the detrimental effects of the flammable gases on the resistance of the sensor layer. This effect is due to the catalytic layer promoting the oxidation of hydrogen and carbon monoxide through the consumption of ambient O_2_ and/or the lattice oxygen atoms of the Pt-CeZr10 catalytic layer.

## 1. Introduction

Industrial gases, such as pure nitrogen, are generally produced by rectification of atmospheric air, during which the concentration of any oxygen present in inert industrial gases should be monitored continually. In terms of quality control, end-users of these inert industrial gases must also monitor the level of oxygen contamination in manufacturing processes. It is therefore crucial that oxygen sensors possess fast-response properties for real-time monitoring. In addition to oxygen, the atmosphere contains small amounts of flammable gases, such as hydrogen which is present in concentrations of approximately 0.5 ppm [1]. Therefore, as there is a possibility that industrial gases can also be contaminated with flammable gases, it is essential that the oxygen sensors are not affected by the presence of flammable gases.

In recent years, a range of oxygen sensors with different detection principles have been developed. In terms of the potential-type [2,3,4,5,6,7,8] and the limiting current-type [9,10,11,12,13,14] sensors, the detection principles involve monitoring of the voltage or current that is generated by the difference in oxygen concentration between both ends of the solid electrolyte. However, the voltage or current of the solid electrolyte is also affected by the flammable gases [3,5,6,13]. The galvanic cell-type, on the other hand, is not affected by the presence of flammable gases [15,16], but unfortunately the sensing response rates of galvanic cell-type sensors are generally too slow for the monitoring processes. The yellow phosphorus luminous-type sensor has therefore been adopted for monitoring, as it has a fast response and is not affected by the presence of flammable gases [17]. Unfortunately, the yellow phosphorus system has a major disadvantage, in that yellow phosphorus is flammable and extremely poisonous, and thus the development of an alternative oxygen sensor system is desired.

We previously reported cerium oxide semiconductor-based resistive oxygen sensors, since cerium oxide is one of the most promising oxygen sensing materials for use in fast-response sensors because of its high diffusion coefficient for oxygen [18,19]. In addition, the cerium oxide-based thick film also has high resistivity, which can be reduced upon doping with 10 mol% zirconium. Indeed, the oxygen sensing response rate of 10 mol% zirconium-doped cerium oxide (Ce_0.9_Zr_0.1_O_2_) is as fast as that of cerium oxide [20]. These resistive oxygen sensors have relatively simple structures, consisting of a cerium oxide-based thick film and a substrate with comb-type electrodes. Therefore, the resistive oxygen sensors can be equipped with additional “covering” layers for the consumption of flammable gases. In this pilot study, we report the development of catalytic layers as the covering layers for the consumption of flammable gases, namely hydrogen and carbon monoxide, in Ce_0.9_Zr_0.1_O_2_ (CeZr10) resistive oxygen sensor layers.

## 2. Experimental Section

### 2.1. Synthesis of CeZr10 Nanoparticles

Cerium nitrate pentahydrate (Kojundo Chemical Laboratory, Sakado, Japan) and zirconyl nitrate dihydrate (Wako Pure Chemical Industries, Osaka, Japan) were dissolved in distilled water to give solutions containing 90 mmol/L Ce^3+^ and 10 mmol/L Zr^4+^, respectively. Aqueous ammonia (25%) was then added dropwise to aqueous solution to give a white precipitate. The resulting suspension was filtered, and the precipitate was mixed with commercialized carbon powder using a HM-500 hybrid mixer (Keyence, Osaka, Japan), to give a precipitate/carbon powder weight ratio of 75:11. The mixture was then dried at 70 °C overnight and annealed at 900 °C for 2 h to give the final product Ce_0.9_Zr_0.1_O_2_ as a fine powder.

### 2.2. Preparation of Thick Film Sensors

Before the preparation of thick films as sensing materials was carried out, a platinum comb-type electrode having a 2.5 × 6.3 mm^2^ area, with a 175 μm gap, and 100 μm line width was formed on a 6.35 × 50.8 mm^2^ alumina substrate and a Pt heater was patterned on the backside of the substrate. 

For preparation of the thick film sensor, the Ce_0.9_Zr_0.1_O_2_ powder was added to an ethylcellulose-type organic dispersant to obtain a paste, with a Ce_0.9_Zr_0.1_O_2_ powder/vehicle weight ratio of 1:4. The Ce_0.9_Zr_0.1_O_2_ paste was then screen-printed onto the electrode of the alumina substrates using an LS-150 screen printer (New Long Seimitsu Kogyo, Tokyo, Japan), dried at 150 °C for 15 min, and the print/dry process repeated four times. The resulting substrate was calcined at 500 °C for 5 h, and fired at 1100 °C for 2 h, to give the Ce_0.9_Zr_0.1_O_2_ thick film sensor, which will be referred to as “CeZr10”.

As per the above process, an additional thick film sensor was prepared, where the print/dry process was repeated eight times, and the substrate was calcined and fired as described above, to give a thick film sensor, which will be referred to as “CeZr10 × 2”.

Layered thick film sensors were then prepared from the obtained CeZr10. Gamma-alumina (γ-alumina) powder, with an average particle size of approximately 100 nm, was prepared by the polyol method, as reported previously [21]. Alumina paste was also prepared using the ethylcellulose-type organic dispersant, with an alumina powder/vehicle weight ratio of 1:2, and was screen-printed onto the CeZr10 thick film. The print/dry process was repeated three times, after which the substrate was dried at 150 °C for 15 min, and calcined at 500 °C for 5 h. The CeZr10 paste was then screen-printed four times onto the alumina thick film, dried at 150 °C for 15 min, and calcined at 800 °C for 2 h. The resulting layered thick film sensor will be referred to as “CeZr10/Al_2_O_3_/CeZr10”.

**Figure 1:**
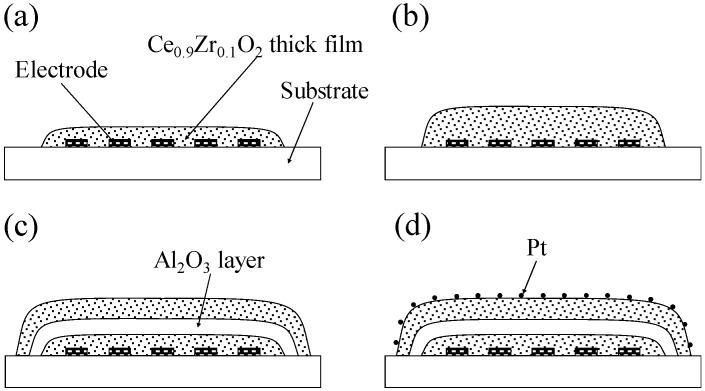
Drawings of cross sections of thick-film sensors: (**a**) CeZr10; (**b**) CeZr10 × 2; (**c**) CeZr10/Al_2_O_3_/CeZr10; (**d**) CeZr10/Al_2_O_3_/Pt-CeZr10.

Finally, layered thick film sensors with added platinum catalyst were prepared from the obtained CeZr10/Al_2_O_3_/CeZr10. A Pt colloidal suspension (particle size: 2 nm, Tanaka Kikinzoku Kogyo K.K., Tokyo, Japan) was added dropwise to the top of the CeZr10 thick film. The resulting Pt content was 3 wt% with respect to the top of the CeZr10 thick film. The resulting thick film with Pt suspension was dried at 70 °C using a hot plate, to give the layered thick film, which will be referred to as “CeZr10/Al_2_O_3_/Pt-CeZr10”. Figure 1 shows drawings of the cross sections of all thick film sensors described herein.

### 2.3. Analysis of the Thick Films

Field emission scanning electron microscopy (FE-SEM) measurements were carried out using a JSM-6355FM microscope (JEOL, Akishima, Japan) to observe the edge sections of the CeZr10/Al_2_O_3_/CeZr10 thick film.

### 2.4. Preparation of Sensor Modules

**Figure 2:**
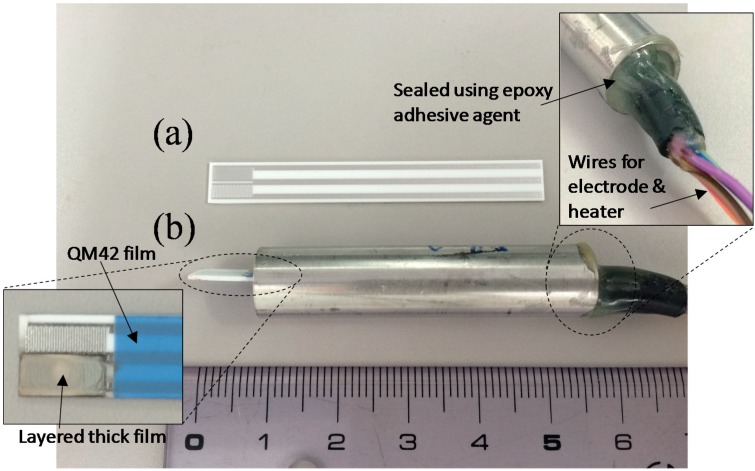
Photograph of (**a**) an alumina substrate with platinum comb-type electrode; and (**b**) a sensor module of a layered thick film sensor using the alumina substrate.

After preparation of the thick film sensors, the insulator paste (QM-42, DuPont, Wilmington, DE, USA) was screen-printed on both the frontside and the backside areas of the substrate, with the exception of the thick film sensor area, in order to cover the Pt electrode lines and the heater. The resulting substrate was dried at 150 °C for 15 min, and calcined at 800 °C for 2 h. The required electrical wires were then connected to the electrode and heater, and the connecting point was covered with thermal shrinkage tube. The resulting sensor elements were inserted into stainless steel tubes of 10 mm diameter, and sealed using an epoxy adhesive agent (AR-R30 Araldite, Nichiban, Japan) to give the final sensor modules, as shown in Figure 2. 

### 2.5. Sensor Response Measurements

The sensor modules were placed in a gas flow stainless steel chamber (approx. 530 mL volume; O-TEC, Tokyo, Japan) and sealed using ferrules for stainless steel tubes (Swagelok, Solon, OH, USA). The sensor modules were heated to 600 °C using Pt heaters, with the resistance of the Pt heater precisely controlled to maintain the sensor modules at a constant temperature. It should be noted that the response rates of the sensor modules were affected by entrapped gas inside the stainless steel tubes, and so the response rates were not investigated in this study.

Gases for analysis including over 3% oxygen were prepared by mixing nitrogen and oxygen, with the concentrations of each being controlled *via* the flow rates of nitrogen and oxygen using a Kofloc 3200 mass flow controller system (Kofloc, Kyoto, Japan), where the total flow rate was maintained at 100 mL/min. The oxygen concentrations used in the study were 100%, 30%, 10%, and 3%, with the concentration being changed every 20 min. 

In the case of ~1 ppm oxygen, the sample gases were prepared from nitrogen (Japan Fine Products; JFP, Kawasaki, Japan), 10 ppm oxygen in nitrogen (JFP), and 10 ppm flammable gases in nitrogen (JFP). These gases are standard gas whose concentration can be traceable to National Institute of Standards and Technology (NIST). The flammable gases used in this study were hydrogen and carbon monoxide. Concentrations of oxygen and flammable gases were precisely controlled by a Stec SEC-4400M mass flow controller system (Horiba, Kyoto, Japan) and the total flow rate was maintained at 1 L/min. After flowing ~0.05 ppm oxygen through the chamber, ~1 ppm oxygen, in either the presence or absence of flammable gases, was flowed for 1–3 h, after which the flow gas was restored back to approximately 0.05 ppm oxygen (except in the case of CeZr10, where the flow gases before and after were composed of 0.05 ppm oxygen with 1 ppm flammable gas). The precise oxygen concentrations of ~0.05 and ~1 ppm were analyzed using a TOA IIS trace oxygen analyzer (Taiyo Nippon Sanso, Tokyo, Japan) whose working principle is based on the yellow phosphorus photoluminescence method [17]. The TOA IIS is accurately-calibrated with standard gas whose concentration is 1 ppm.

### 2.6. Conversion of the Resistance Measurements to Oxygen Concentration Values

The resistance measured for each sample gas was converted into oxygen concentration in order to evaluate the accuracy of the sensor modules. The resistances were substituted into a linear regression equation in order to convert the resistance into oxygen concentration (such as the equation for CeZr10/Al_2_O_3_/Pt-CeZr10, as shown in Figure 6f below). The equations were obtained from the dataset of resistances under several oxygen concentrations (100%, 30%, 10%, 3%, ~1 ppm, and ~0.05 ppm), without the presence of flammable gases. In the case of ~0.05 and ~1 ppm oxygen, the precise oxygen concentrations analyzed by means of the Taiyo Nippon Sanso TOA IIS were used for the equations.

## 3. Results and Discussion

### 3.1. Effects of an Alumina Thick Film as an Insulator

**Figure 3:**
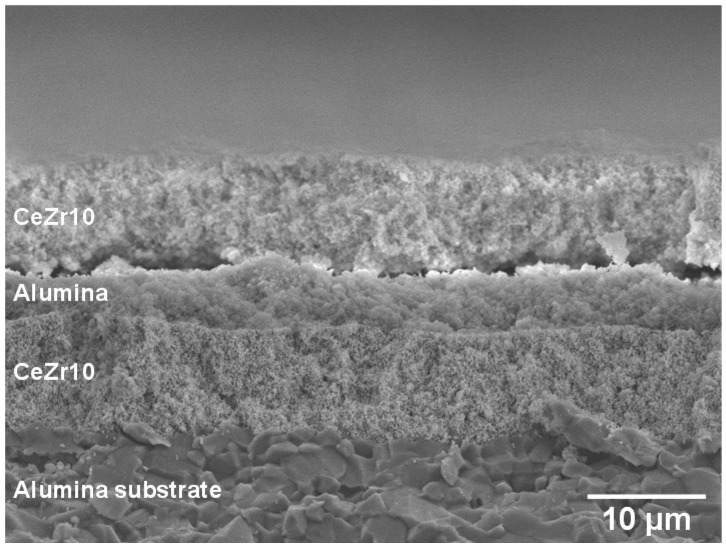
Cross-sectional FE-SEM image of the CeZr10/Al_2_O_3_/CeZr10 layered thick film.

Figure 3 shows a cross-sectional FE-SEM image of the CeZr10/Al_2_O_3_/CeZr10 layered thick film. Borders between the lower CeZr10 layer and the middle Al_2_O_3_ layer, and between the Al_2_O_3_ layer and the upper CeZr10 layer are clearly defined, and it is clear that the two CeZr10 layers are successfully separated by the middle Al_2_O_3_ layer. It can also be seen that the thickness of both the upper and lower CeZr10 layers is more or less equivalent, with the thicknesses of CeZr10 and Al_2_O_3_ layers being approximately 9 and 4 μm, respectively. 

**Figure 4:**
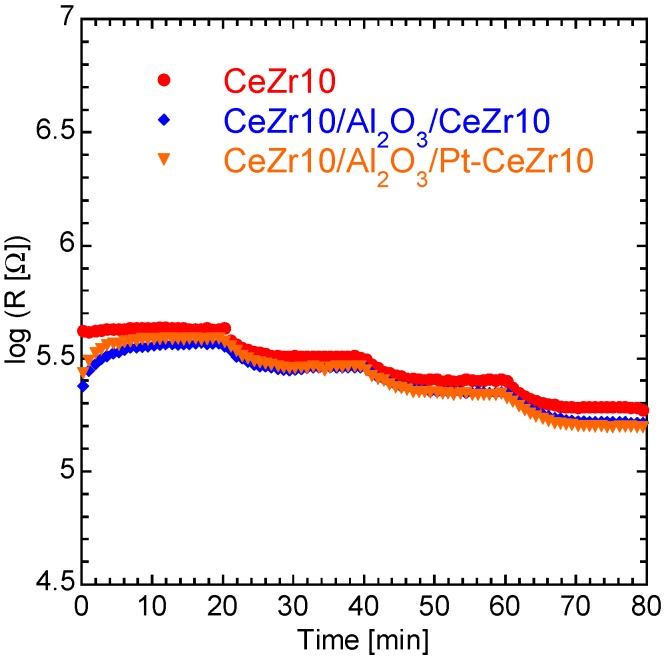
Dynamic resistance response of CeZr10, CeZr10/Al_2_O_3_/CeZr10, and CeZr10/Al_2_O_3_/Pt-CeZr10 thick films to oxygen. The concentrations of oxygen used were 100, 30, 10, and 3% in nitrogen.

Figure 4 shows the dynamic resistance response of CeZr10, CeZr10/Al_2_O_3_/CeZr10, and CeZr10/Al_2_O_3_/Pt-CeZr10 thick films in the presence of >3% oxygen. It can be seen from this plot that the sensor films having catalytic layer, namely CeZr10/Al_2_O_3_/CeZr10 and CeZr10/Al_2_O_3_/Pt-CeZr10, show comparable resistance profiles to that of CeZr10. 

**Figure 5:**
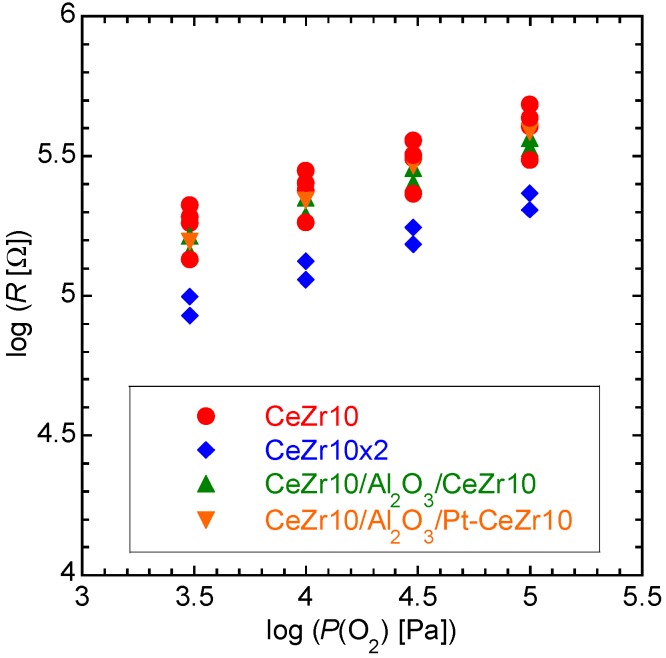
Resistance of CeZr10, CeZr10 × 2, CeZr10/Al_2_O_3_/CeZr10, and CeZr10/Al_2_O_3_/Pt-CeZr10 in the presence of >3% oxygen.

As previously described, the catalytic layers, namely CeZr10 and a Pt-loaded CeZr10 layer, were composed of the same material as the lower sensor layer (*i.e.*, CeZr10). The electrical resistance of the CeZr10 layer depends mainly on the oxygen partial pressure, as the concentration of oxygen vacancies present in the CeZr10 layers depends on the partial pressure of oxygen, and also because of the relationship between the electrical resistance and the oxygen vacancy concentration. Flammable gas molecules present in the CeZr10 system, *i.e*., hydrogen and carbon monoxide, are oxidized with the consumption of lattice oxygen from the CeZr10 layers, and so the electrical resistance of the CeZr10 layers is also affected by the presence of flammable gases. Therefore, the lower CeZr10 thick film sensor layer should be insulated from the upper CeZr10 or Pt-CeZr10 catalytic layer by the use of a middle insulating layer, in this case, Al_2_O_3_. Figure 5 shows the resistances of CeZr10, CeZr10 × 2, CeZr10/Al_2_O_3_/CeZr10, and CeZr10/Al_2_O_3_/Pt-CeZr10 in the presence of >3% oxygen. The oxygen concentrations were converted to logarithms of oxygen partial pressures. For example, the logarithms of oxygen partial pressures for 3% and 100% oxygen are 3.48 and 5.00, respectively. Although discrepancies exist in the resistance observed from a number of CeZr10 and CeZr10 × 2 systems, we could calculate that the resistance of CeZr10 × 2 is approximately half that of CeZr10. In addition, the film thickness of CeZr10 × 2 was estimated to be twice that of CeZr10, due to the fact that the CeZr10 × 2 layer was prepared using twice the number of print/dry processes compared to CeZr10. This led to the conclusion that increasing the film thickness results in a decrease in the electric resistance of the CeZr10-based thick film system. The resistances obtained for both CeZr10/Al_2_O_3_/CeZr10 and CeZr10/Al_2_O_3_/Pt-CeZr10 were comparable to those of CeZr10. It could therefore be concluded that the middle Al_2_O_3_ layer does indeed function as an insulating layer between the lower CeZr10 oxygen sensor layer and the upper CeZr10 or Pt-CeZr10 catalytic layer.

### 3.2. Sensing Properties in Low Oxygen Concentrations

**Figure 6:**
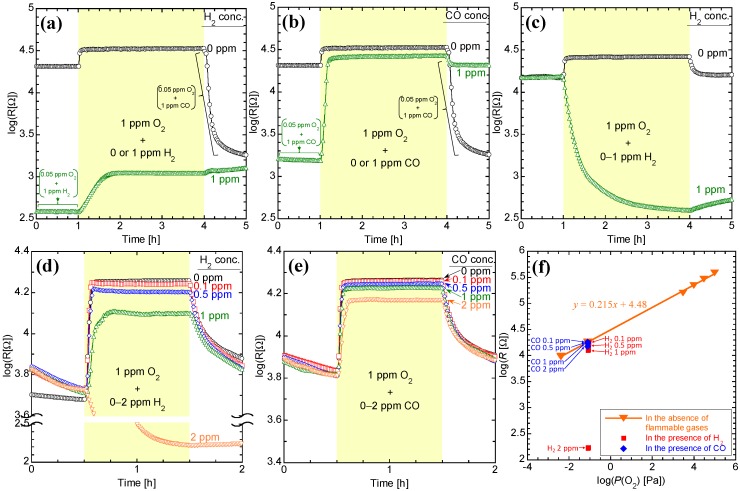
Dynamic resistance response of (**a**,**b**) CeZr10; (**c**) CeZr10/Al_2_O_3_/CeZr10; and (**d**,**e**) CeZr10/Al_2_O_3_/Pt-CeZr10 to 1 ppm oxygen in the presence of flammable gases (**a**,**c**,**d** = hydrogen; **b**,**e** = carbon monoxide); (**f**) the linear regression equation from resistances of CeZr10/Al_2_O_3_/Pt-CeZr10 for the converted oxygen concentrations. The resistances in ~1 ppm oxygen are plotted from Figure 6d,e.

Figure 6 shows the dynamic resistance responses of thick film sensors to a gas flow containing 1 ppm oxygen, both in the presence and absence of flammable gases, while Table 1 shows the resistances and converted oxygen concentrations of the sensors. The converted oxygen concentrations were obtained from the linear regression equation, as previously described in Section 2.6 and as indicated in Figure 6f for the CeZr10/Al_2_O_3_/Pt-CeZr10 system. The converted oxygen concentrations in a gas flow of ~1 ppm oxygen in the absence of flammable gases were also obtained from the resistance values, by comparing to the results in 1 ppm oxygen in the presence of flammable gases. 

As shown in Table 1, the converted oxygen concentration of CeZr10 was calculated to be 0.61 ppm in the absence of flammable gases. In addition, the resistance of CeZr10 dropped significantly in the presence of 1 ppm hydrogen, as shown in Figure 6a and Table 1. As the oxidation of one mole of H_2_ requires only half a mole of O_2_, the converted oxygen concentration of CeZr10 in 1 ppm O_2_ and H_2_ would be 0.11 ppm, even if the oxidation of 1 ppm H_2_ consumed ambient O_2_. 

**Table 1:** Resistances and converted oxygen concentrations of thick film sensors in ~1 ppm oxygen, in the presence and absence of flammable gases.

Flammable Gas		CeZr10		CeZr10/Al_2_O_3_/CeZr10		CeZr10/Al_2_O_3_/Pt-CeZr10	
	Conc. (ppm)	log(*R* (Ω)) ^1)^	Conv. O_2_ Conc. (ppm) ^2)^	log(*R* (Ω)) ^1)^	Conv. O_2_ Conc. (ppm) ^2)^	log(*R* (Ω)) ^1)^	Conv. O_2_ Conc. (ppm) ^2)^
-	0	4.55	0.61	4.42	0.66	4.26	0.88
H_2_	0.1	-	-	-	-	4.25	0.85
	0.5	-	-	-	-	4.22	0.57
	1	3.04	5.7 × 10^−10^	2.60	5.8 × 10^−11^	4.11	0.19
	2	-	-	-	-	2.23	3.5 × 10^−10^
CO	0.1	-	-	-	-	4.26	0.87
	0.5	-	-	-	-	4.25	0.78
	1	4.43	0.11	-	-	4.23	0.64
	2	-	-	-	-	4.17	0.36

^1)^ Logarithm of resistance; ^2)^ Converted oxygen concentration.

However, the converted oxygen concentration of CeZr10 in 1 ppm O_2_ and H_2_ was found to be much lower (5.7 × 10^−10^ ppm). These results indicate that the oxidation of H_2_ took place through the consumption of oxygen atoms from CeZr10, and thus induced the formation of oxygen vacancies. 

Unlike the standard CeZr10 system, the CeZr10/Al_2_O_3_/CeZr10 module contains an upper CeZr10 catalytic layer. However, the resistance of CeZr10/Al_2_O_3_/CeZr10 decreased significantly in the presence of 1 ppm hydrogen, as shown in Figure 6c, and the converted oxygen concentration of CeZr10/Al_2_O_3_/CeZr10 was low (Table 1). In this system, the upper CeZr10 catalytic layer should not be capable of oxidizing all H_2_ molecules, and therefore a proportion of the H_2_ molecules must reach the lower CeZr10 sensor layer and be oxidized by lattice oxygen atoms from this layer.

The addition of platinum to the upper CeZr10 catalytic layer, to give CeZr10/Al_2_O_3_/Pt-CeZr10, was expected to beneficial in preventing the negative effects of flammable gases. The decrease in resistance observed in the presence of 1 ppm of O_2_ and H_2_ was so small that the converted oxygen concentrations were sub-ppm levels. This indicates that the Pt additive promotes the oxidation of H_2_ through the consumption of ambient O_2_ and/or lattice oxygen atoms of the upper Pt-CeZr10 catalytic layer. However, the decrease in converted oxygen concentration was greater than half of the H_2_ concentration when the H_2_ concentration was greater than 0.5 ppm, as shown in Table 1. Therefore, the catalytic Pt-CeZr10 layer should not consume all H_2_ molecules, allowing some to reach the lower CeZr10 layer and become oxidized by the lattice oxygen atoms. Indeed, as shown in Figure 6d, the resistance showed a sharp and significant decrease in the presence of 2 ppm H_2_ gas.

The converted oxygen concentration of CeZr10/Al_2_O_3_/Pt-CeZr10 in the presence of 1 ppm O_2_ and CO was also higher than that of CeZr10, as shown in Table 1 and Figure 6e. In the case of CeZr10, the decrease in converted oxygen concentrations were approximately half that of the CO concentrations, demonstrating that the CeZr10 layer provides few lattice oxygen atoms for the oxidation of CO. However, in the case of CeZr10/Al_2_O_3_/Pt-CeZr10, the decrease in converted oxygen concentrations was less than half that of the CO concentrations. This further demonstrates that the addition of platinum to upper CeZr10 catalytic layer activates the supply of lattice oxygen atoms in the upper CeZr10 layer for the oxidation of CO.

## 4. Conclusions

We successfully prepared four kinds of layered sensor thick films, namely CeZr10, CeZr10 × 2, CeZr10/Al_2_O_3_/CeZr10, and CeZr10/Al_2_O_3_/Pt-CeZr10. In the case of CeZr10/Al_2_O_3_/CeZr10, we confirmed that the upper CeZr10 layer was not in contact with the lower CeZr10 layer because of the presence of the middle Al_2_O_3_ layer. The CeZr10/Al_2_O_3_/CeZr10 and CeZr10/Al_2_O_3_/Pt-CeZr10 modules showed resistance profiles comparable to that of CeZr10, while the resistance of CeZr10 × 2 was approximately half that of CeZr10. This demonstrated that the middle Al_2_O_3_ layer behaves as an insulating layer between the lower CeZr10 layer and the upper CeZr10 or Pt-CeZr10 layer. With an oxygen concentration of approximately 1 ppm, the resistance of CeZr10 and CeZr10/Al_2_O_3_/CeZr10 decreased significantly when 1 ppm of hydrogen was included in the gas mixture. This occurred because the oxidation of hydrogen consumed oxygen atoms from the lower CeZr10 oxygen sensor layer. The converted oxygen concentrations obtained from the resistance values were extremely small for both CeZr10 and CeZr10/Al_2_O_3_/CeZr10, *i.e.*, 5.7 × 10^−10^ and 5.8 × 10^−11^ ppm, respectively. In addition, the resistance of CeZr10 also showed a slight decrease in the presence of 1 ppm of CO, giving a converted oxygen concentration of 0.11 ppm (CeZr10), owing to the oxidation of carbon monoxide by ambient O_2_. 

The addition of platinum to the upper CeZr10 catalytic layer to give CeZr10/Al_2_O_3_/Pt-CeZr10 was expected to prevent the described effects of hydrogen and carbon monoxide. This occurred as the modified upper catalytic layer promoted the oxidation of hydrogen and carbon monoxide through consumption of ambient O_2_ and/or lattice oxygen atoms from the upper Pt-CeZr10 catalytic layer, rather than the lower oxygen-sensing layer. Indeed, the Pt-CeZr10 catalytic layer showed good performance for protecting the CeZr10 thick film oxygen-sensing layer from the effects of flammable gases. However, the performance of the current Pt-CeZr10 catalytic layer is insufficient for monitoring low concentrations of O_2_ in the presence of flammable gases. The investigation of catalytic supports, which can supply lattice oxygen atoms for the oxidation of flammable gas molecules, will be conducted in the future.
